# Supplementary material for: Transcriptome analysis of substrate temperature effects on adventitious root formation in peach rootstocks
Source: PeerJ. 2025 Sep 5;13:e20015. doi: 10.7717/peerj.20015 (PMC12422280; doi:10.7717/peerj.20015)
Supplement: Supplemental Information 2 [file peerj-13-20015-s002.doc]

Supplementary file 1. Sample comparison area statistical results.

| Group | sample | multiple mapped | unique mapped | exon | intron | intergenic |
| --- | --- | --- | --- | --- | --- | --- |
| CT0 | CK1 | 876108(1.86%) | 42491770(90.37%) | 6289213416(96.8190%) | 133423894(2.0540%) | 73208150(1.1270%) |
|  | CK2 | 903803(1.92%) | 42620725(90.58%) | 6326316209(97.0396%) | 118092277(1.8114%) | 74902023(1.1489%) |
|  | CK3 | 889642(1.87%) | 42690083(89.89%) | 6324912745(96.9001%) | 128602666(1.9702%) | 73740461(1.1297%) |
| CT1 | T1_1 | 856061(1.89%) | 40845269(90.16%) | 6042437925(96.7404%) | 99779820(1.5975%) | 103813530(1.6621%) |
|  | T1_2 | 878781(1.92%) | 41110298(89.9%) | 6099629281(96.9890%) | 95988970(1.5263%) | 93371347(1.4847%) |
|  | T1_3 | 722415(1.54%) | 40093942(85.5%) | 5930156918(97.1255%) | 67878207(1.1117%) | 107633776(1.7628%) |
| CT2 | T2_1 | 793749(1.77%) | 40574997(90.32%) | 6016568645(97.0984%) | 96123160(1.5513%) | 83672884(1.3504%) |
|  | T2_2 | 722104(1.79%) | 36466377(90.22%) | 5410156003(97.1291%) | 80811284(1.4508%) | 79099629(1.4201%) |
|  | T2_3 | 848911(1.81%) | 42390395(90.4%) | 6282300958(97.0008%) | 99147596(1.5309%) | 95096249(1.4683%) |
| CT3 | T3_1 | 860778(1.78%) | 43459768(89.7%) | 6455736310(97.2512%) | 98273951(1.4804%) | 84197414(1.2684%) |
|  | T3_2 | 755342(1.77%) | 38920203(91.1%) | 5769788292(97.0853%) | 99228129(1.6697%) | 73996733(1.2451%) |
|  | T3_3 | 804832(1.75%) | 41917974(90.9%) | 6199493865(96.8720%) | 116883061(1.8264%) | 83298612(1.3016%) |
| CT4 | T4_1 | 822674(1.76%) | 41797889(89.67%) | 6177573010(96.7705%) | 106879170(1.6742%) | 99280085(1.5552%) |
|  | T4_2 | 710216(1.74%) | 36783449(90.33%) | 5450379123(97.0498%) | 80578681(1.4348%) | 85108438(1.5154%) |
|  | T4_3 | 814908(1.77%) | 41465327(89.88%) | 6139321824(96.9478%) | 112711829(1.7799%) | 80573980(1.2724%) |
| CT5 | T5_1 | 734181(1.76%) | 37631947(90.1%) | 5568361573(96.8942%) | 95781306(1.6667%) | 82704513(1.4391%) |
|  | T5_2 | 694857(1.75%) | 35457214(89.24%) | 5243232300(96.8348%) | 91222991(1.6848%) | 80162179(1.4805%) |
|  | T5_3 | 818454(1.74%) | 42067211(89.54%) | 6198517601(96.4964%) | 108457981(1.6884%) | 116593962(1.8151%) |
| CT6 | CK_14_1 | 835349(1.86%) | 40065257(89.32%) | 5862942381(95.7046%) | 120436648(1.9660%) | 142698865(2.3294%) |
|  | CK_14_2 | 779848(1.8%) | 38667313(89.14%) | 5639308277(95.4476%) | 119329168(2.0197%) | 149638547(2.5327%) |
|  | CK_14_3 | 864242(1.8%) | 42667541(88.86%) | 6228804149(95.5300%) | 128653810(1.9731%) | 162807330(2.4969%) |
| CT7 | T6_1 | 772347(1.76%) | 39075424(89.29%) | 5715847167(95.7676%) | 113693665(1.9049%) | 138915936(2.3275%) |
|  | T6_2 | 820373(1.75%) | 41834975(89.45%) | 6110587768(95.6397%) | 137675651(2.1548%) | 140910535(2.2055%) |
|  | T6_3 | 768373(1.75%) | 39268522(89.46%) | 5774478874(96.2924%) | 105498294(1.7592%) | 116838965(1.9483%) |
| CT8 | T7_1 | 707604(1.76%) | 36008311(89.47%) | 5299436668(96.3648%) | 99651801(1.8121%) | 100261253(1.8231%) |
|  | T7_2 | 685879(1.72%) | 36024332(90.11%) | 5320957397(96.7691%) | 95833032(1.7429%) | 81823928(1.4881%) |
|  | T7_3 | 701833(1.76%) | 35848864(90.09%) | 5285691476(96.5467%) | 97949889(1.7891%) | 91108433(1.6642%) |
| CT9 | T8_1 | 851680(1.84%) | 41265544(89.08%) | 6078624024(96.3587%) | 122384305(1.9400%) | 107324609(1.7013%) |
|  | T8_2 | 735761(1.81%) | 36340672(89.34%) | 5348553440(96.3128%) | 106914493(1.9252%) | 97850254(1.7620%) |
|  | T8_3 | 800212(1.83%) | 39025442(89.11%) | 5739789745(96.2208%) | 110588620(1.8539%) | 114847538(1.9253%) |
| CT10 | T9_1 | 791674(1.8%) | 39379757(89.69%) | 5766014261(95.8295%) | 118619896(1.9714%) | 132318382(2.1991%) |
|  | T9_2 | 776536(1.8%) | 38759911(89.81%) | 5673016246(95.7948%) | 125242415(2.1148%) | 123792839(2.0904%) |
|  | T9_3 | 688018(1.76%) | 35172983(90.1%) | 5161466042(96.0872%) | 104792467(1.9508%) | 105390561(1.9620%) |
| CT11 | CK_21_1 | 856014(1.9%) | 39995268(88.73%) | 5865754432(95.8675%) | 125347054(2.0486%) | 127505189(2.0839%) |
|  | CK_21_2 | 883604(1.91%) | 41212587(89.2%) | 6065685446(96.2026%) | 125876283(1.9964%) | 113555236(1.8010%) |
|  | CK_21_3 | 725325(1.81%) | 35619380(89.11%) | 5252189511(96.4802%) | 99793612(1.8332%) | 91820858(1.6867%) |
| CT12 | T10_1 | 856471(1.81%) | 42165437(89.03%) | 6181921824(95.9341%) | 142999638(2.2191%) | 119006135(1.8468%) |
|  | T10_2 | 840037(1.81%) | 41173512(88.87%) | 6044235363(96.0515%) | 133388082(2.1197%) | 115082425(1.8288%) |
|  | T10_3 | 878497(1.81%) | 43280512(89.13%) | 6353867307(96.0635%) | 144103747(2.1787%) | 116270719(1.7579%) |
| CT13 | T11_1 | 844653(1.78%) | 42411394(89.57%) | 6194051251(95.5977%) | 148773120(2.2961%) | 136466529(2.1062%) |
|  | T11_2 | 843547(1.78%) | 42130009(88.69%) | 6161065686(95.7244%) | 147615027(2.2935%) | 127572118(1.9821%) |
|  | T11_3 | 801171(1.77%) | 40472941(89.53%) | 5915368807(95.6826%) | 124450571(2.0130%) | 142464043(2.3044%) |
| CT14 | T12_1 | 796061(1.91%) | 36166618(86.77%) | 5314397717(95.9858%) | 121752873(2.1990%) | 100497305(1.8151%) |
|  | T12_2 | 789731(1.65%) | 39351230(82.1%) | 5754902444(95.7156%) | 157577766(2.6208%) | 100019045(1.6635%) |
|  | T12_3 | 796129(1.93%) | 36547992(88.81%) | 5361795040(95.8570%) | 142899783(2.5547%) | 88838589(1.5882%) |
| CT15 | T13_1 | 852603(1.83%) | 40454250(86.78%) | 5873016087(94.9458%) | 206837479(3.3438%) | 105794062(1.7103%) |
|  | T13_2 | 845900(1.81%) | 40979343(87.85%) | 5961352989(95.1658%) | 196717792(3.1404%) | 106103556(1.6938%) |
|  | T13_3 | 828349(1.84%) | 39803545(88.46%) | 5770211858(94.8164%) | 200061055(3.2874%) | 115397386(1.8962%) |
