# Supplementary figures and images for: Transcriptome analysis of substrate temperature effects on adventitious root formation in peach rootstocks
Source: PeerJ. 2025 Sep 5;13:e20015. doi: 10.7717/peerj.20015 (PMC12422280; doi:10.7717/peerj.20015)

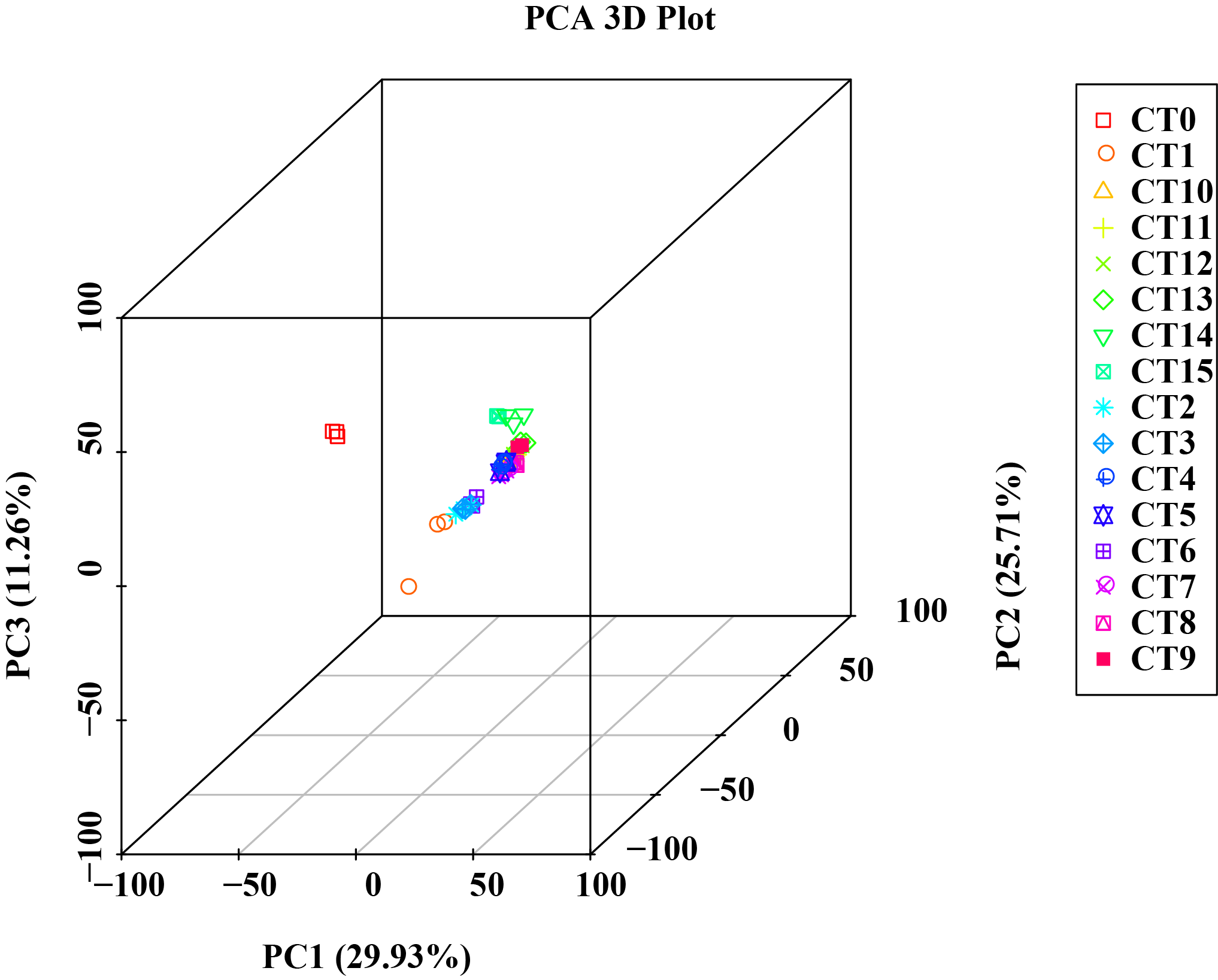

Supplement: Supplemental Information 4 [file peerj-13-20015-s004.png]

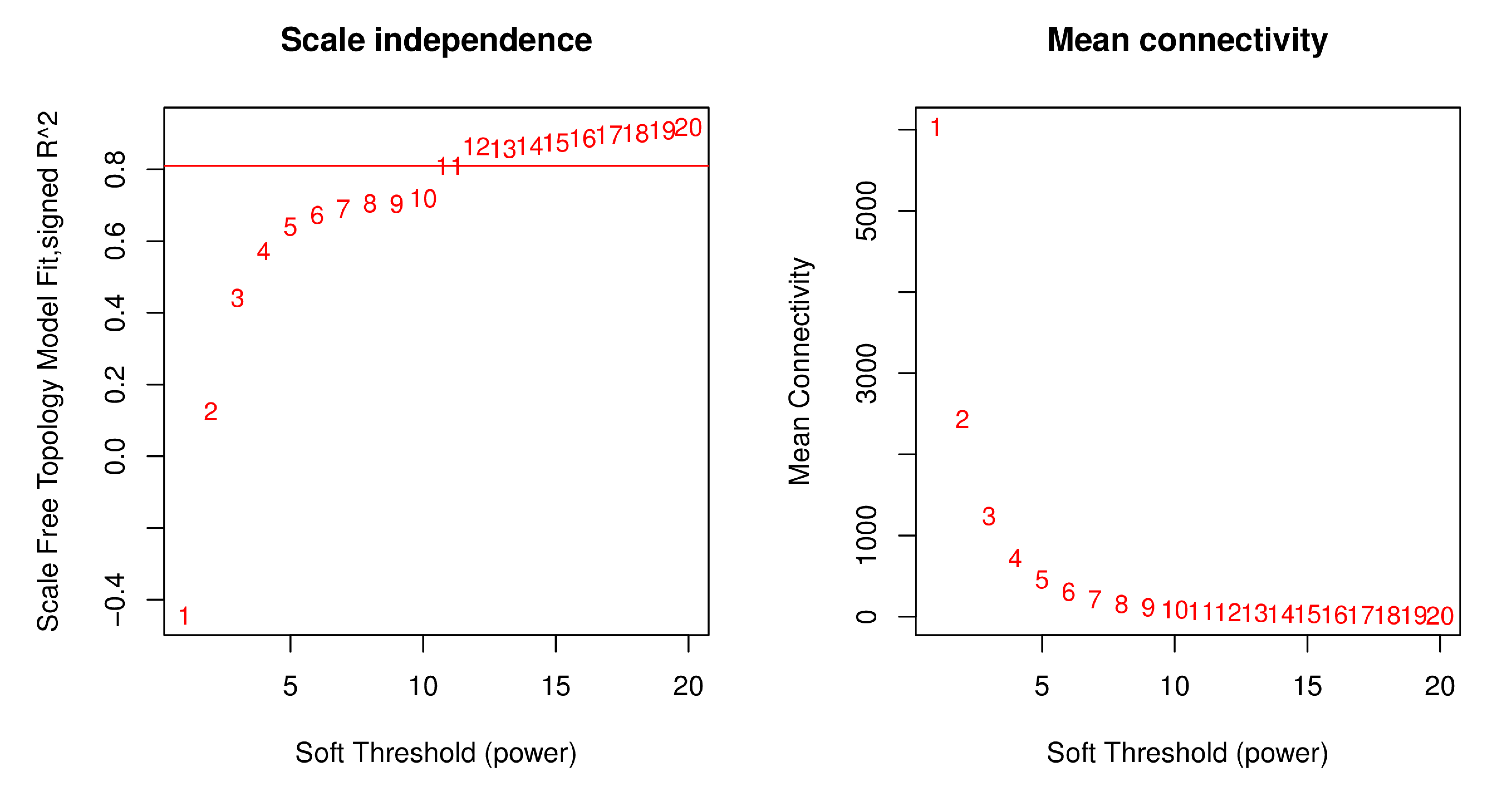

Supplement: Supplemental Information 5 — Soft Threshold (power) represents different soft thresholds, ranging from 1 to 20. Scale Free Topology Model Fit, signed R2 represents the fitting index of the scale-free network. Mean Connectivity refers to the average number of connections for each node in the network. [file peerj-13-20015-s005.png]
