# Supplementary material for: Transcriptome analysis of substrate temperature effects on adventitious root formation in peach rootstocks
Source: PeerJ. 2025 Sep 5;13:e20015. doi: 10.7717/peerj.20015 (PMC12422280; doi:10.7717/peerj.20015)
Supplement: Supplemental Information 6 [file peerj-13-20015-s006.doc]

Supplementary file 5. Transcription factors associated with adventitious root formation.

| Transcription factor | Gene-module | gene_id |
| --- | --- | --- |
| WRKY | plum | 18782441 |
|  | darkolivegreen | 109947108 |
|  | darkolivegreen | 18783428 |
|  | paleturquoise | 18792641 |
| ERF | plum | 109946487 |
|  | mediumorchid | 18776717 |
|  | darkolivegreen | 18776669 |
|  | darkolivegreen | 18782299 |
|  | paleturquoise | 18768424 |
|  | darkolivegreen | 18781384 |
|  | darkolivegreen | 18771426 |
|  | darkolivegreen | 109949018 |
| NAC | plum | 18769080 |
|  | plum | 109946542 |
|  | plum | 18785642 |
|  | mediumorchid | 18773403 |
|  | mediumorchid | 18781029 |
|  | mediumorchid | 109949876 |
| bHLH | plum | 18776062 |
|  | mediumorchid | 109950855 |
|  | mediumorchid | 18785877 |
|  | mediumorchid | 18785223 |
|  | darkolivegreen | 18767454 |
|  | darkolivegreen | 18778547 |
|  | darkolivegreen | 18771109 |
|  | darkolivegreen | 18766566 |
|  | paleturquoise | 18781354 |
|  | paleturquoise | 18773682 |
|  | paleturquoise | 18782064 |
| bZIP | mediumorchid | 18778892 |
|  | darkolivegreen | 18785809 |
|  | darkolivegreen | 109947329 |
|  | paleturquoise | 18772793 |
|  | paleturquoise | 18786101 |
| MYB | plum | 18785749 |
|  | mediumorchid | 18790681 |
|  | plum | 18786636 |
|  | plum | 18792968 |
|  | darkolivegreen | 18776109 |
|  | darkolivegreen | 109949164 |
|  | darkolivegreen | 109950038 |
|  | darkolivegreen | 109949171 |
|  | paleturquoise | 18785999 |
|  | paleturquoise | 18787158 |
|  | paleturquoise | 109946543 |
|  | paleturquoise | 18785361 |
|  | paleturquoise | 18784022 |
|  | darkolivegreen | 18783732 |
